# The Effects of Temperature and Ethanol on Proanthocyanidin Adsorption to Grape Cell Wall Material in the Presence of Anthocyanins

**DOI:** 10.3390/molecules25184139

**Published:** 2020-09-10

**Authors:** Jordan W. Beaver, Konrad V. Miller, Cristina Medina-Plaza, Nick Dokoozlian, Ravi Ponangi, Thomas Blair, David Block, Anita Oberholster

**Affiliations:** 1 Department of Viticulture and Enology, University of California, One Shields Avenue, Davis, CA 95616, USA; vonmiller@ucdavis.edu (K.V.M.); cmedinaplaza@ucdavis.edu (C.M.-P.); deblock@ucdavis.edu (D.B.); 2 Department of Chemistry and Biochemistry, University of Texas at Tyler, 3900 University Blvd, Tyler, TX 75707, USA; 3 E&J Gallo Winery, 600 Yosemite Blvd, Modesto, CA 95354, USA; nick.dokoozlian@ejgallo.com (N.D.); ravi.ponangi@ejgallo.com (R.P.); tom.blair@ejgallo.com (T.B.)

**Keywords:** proanthocyanidins, anthocyanins, adsorption, isotherm, modeling, grape, wine, cell wall material, kinetics

## Abstract

The quantitative and qualitative impacts of anthocyanins on proanthocyanidin adsorption to grape-derived cell wall material were investigated in fifteen unique systems of varying temperatures, ethanol concentrations, and proanthocyanidin concentrations. Proanthocyanidin solutions were exposed to cell wall material and monitored for changes in concentration over 24 h. Increases in both temperature and ethanol resulted in a larger retention of proanthocyanidins in solution and typically faster adsorption kinetics. Analysis of the solution after exposure to cell wall revealed a significant reduction in the molecular weight of proanthocyanidins present in solution, suggesting that anthocyanins do not alter a previously described mechanism of preferentially binding large molecular weight molecules. Additionally, a reduction in polymeric pigment abundance was noted in most conditions, suggesting rapid formation of polymeric pigment in the model solution and preferential adsorption of the polymeric pigment to cell wall material. Compared to a previous study of proanthocyanidin adsorption in the absence of anthocyanins, a significantly larger percentage of proanthocyanidin material was lost via adsorption—up to 70% of available material. In a winemaking context, this may suggest a preferential loss of polymeric pigment via adsorption to cap cell wall material compared to non-pigmented proanthocyanidins and free anthocyanins.

## 1. Introduction

It is well established that proanthocyanidins (PAs) play an important role in both the development of grapes and the production of wine. In the berries of *Vitis vinifera*, PAs are synthesized via the flavonoid pathway and are believed to serve primarily as a phytochemical response to mechanical or biological stresses [1]. Localized to the vacuoles of skin and seed cells, PAs are released during the crushing process, along with other key polyphenols, as the vacuoles of the cells are ruptured. A portion of these polyphenols are immediately solubilized in the juice, but the majority are extracted into the must/wine matrix over time during the maceration process [2]. PAs have a significant impact on the taste, mouthfeel, color, and oxidative stability of red wine, thus high extractability is generally desired during the production process [3]. However, it has been shown that PA extractability is significantly hindered by adsorption interactions with solids in the fermenter [4].

Previous studies have detailed PA adsorption during red wine fermentations to some degree. Bindon et al. [5] revealed the extent to which the cell wall material (CWM) of grape marc can selectively remove PA from model wine solutions; nearly 60% of available PAs were lost to adsorption. It has been proposed that the types of interactions between PA and CWM are hydrogen bonding, hydrophobic interactions, and Van Der Waals forces [6]. In the context of wine production, however, there are many other components within the wine matrix that likely impact PA adsorption. Previous studies have detailed the impact of temperature and ethanol on PA–CWM interactions, showing a synergistic impact on PA remaining in solution after initial contact and the recovery of adsorbed PA as temperature and ethanol increase [7,8,9]. Additionally, the presence of anthocyanins has been suggested to influence PA extractability. However, separate studies that investigated this impact in winemaking trials showed conflicting results regarding whether anthocyanins increased or decreased PA adsorption [10,11]. A 2016 study by Bautista-Ortin et al. [12] investigated tannin adsorption in the presence and absence of anthocyanins in a model wine system at constant temperature (25 °C) and ethanol (12% *v*/*v*). Results showed that the presence of anthocyanins significantly reduced the adsorption of PA to CWM, suggesting that flavonoids compete for available adsorption sites on the CWM. Considering that the composition and conformation of most cell wall material induce a negative surface charge and that flavylium anthocyanins contain a cationic oxygen on their C-ring, it would stand to reason that the PA–CWM interactions may be disrupted by the presence of the charged anthocyanins. The anthocyanin–CWM interactions would likely be more thermodynamically favorable by comparison to PA–CWM interactions [13,14]. However, the study by Bautista-Ortin et al. only studied this phenomenon under a single temperature, ethanol concentration, and PA concentration, all of which could potentially influence the adsorption mechanism or even compositional and conformational changes in PA molecules [12]. Similar to PA behavior, a recent study by Medina-Plaza et al. [14] shows a synergistic influence of temperature and ethanol concentration on isolated anthocyanin adsorption to grape-derived CWM in model wines. By comparison, the kinetics of anthocyanin adsorption to CWM were shown to be much faster than PA adsorption [7].

Considering the synergistic influence of temperature and ethanol on both PA and anthocyanin adsorption to CWM, it is important to understand how adsorption interactions with CWM is altered under varying winemaking conditions. The following study utilizes a factorial design of different PA concentrations, environmental temperatures, and ethanol concentrations to investigate the PA–CWM interactions in the presence of anthocyanins. The adsorption kinetics, quantitative adsorption, and compositional changes of PA in solution will be presented and compared to previous work that detailed PA–CWM interactions in the absence of anthocyanins [7]. This comparison may help to elucidate the mechanistic impacts of anthocyanins on these adsorption interactions and further our understanding of polyphenol extraction in red wine fermentations.

## 2. Results

### 2.1. Proanthocyanidin Progress Curves

**Figure 1:**
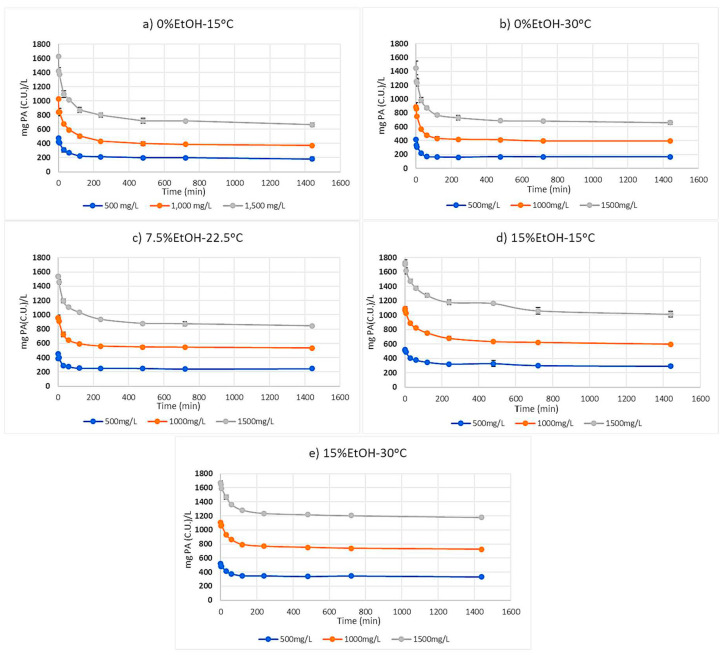
Progress curves of average proanthocyanidin (PA) concentration in solution after exposure to cell wall material (CWM). Standard deviation bars are displayed but often too small to be seen. (**a**) 0% ethanol MW, 15 °C incubation; (**b**) 0% ethanol MW, 30 °C incubation; (**c**) 7.5% ethanol MW, 22.5 °C incubation; (**d**) 15% ethanol MW, 15 °C incubation; (**e**) 15% ethanol MW, 30 °C incubation (*n* = 3 for each point on progress curves; *p* < 0.05).

Progress curves showing the reduction in PA concentration in the solution for each condition are displayed in Figure 1. Trials are labeled by their approximate starting concentration of PA material (500, 1000, and 1500 mg/L), and conditions are labeled by their ethanol concentration of model wine and incubation temperature. Concentration of the PA solution notably decreased in all trials, but the extent of adsorption and the equilibration time (i.e., showing no significant change in PA concentration in solution) varied for each condition too. In all conditions, an initial rapid adsorption was noted within the first 120 min which is consistent with previous studies [5,7]. It should be noted that an initial adsorption trial was performed over 2880 min (data not shown). No significant change in PA concentration was ever noted between 1440 and 2880 min, thus the experimental duration was modified to this time point.

In the 0%EtOH-15 °C conditions containing 1000 and 1500 mg/L PA material (Figure 1a), loss of PA from solution continued until the 1440-minute sample. This suggests that the PA concentration continues to play a role in the reaction kinetics of CWM interactions. Equilibrium for the 500 mg/L condition was noted after 480 min. The average PA concentration in the 500 mg/L condition did show a slight decrease between the 480- and 1440-min samples, but these values were not significantly different from one another.

Under the 0%EtOH-30 °C condition (Figure 1b), only the 1500 mg/L condition continued to show significant PA reduction in solution up to the 1440-minute mark. The 500 mg/L and 1000 mg/L PA conditions were equilibrated by 120 and 480 min, respectively. The increase in temperature displayed faster equilibration times except for the 1500 mg/L PA condition.

In the 7.5%EtOH-22.5 °C system (Figure 1c), noted equilibration times for the 500 mg/L, 1000 mg/L, and 1500 mg/L PA were 240, 480, and 1440 min, respectively. These results continue to suggest a synergistic impact of PA concentration and temperature as well as ethanol concentration on the adsorption kinetics between PA and the CWM.

Under the conditions of lower temperature (15 °C) and high ethanol concentration (15%), the impact of ethanol on PA adsorption kinetics is highlighted (Figure 1d). Compared to 1440 min in the 0%EtOH-15 °C trials, noted equilibration times for the 1000 mg/L and 1500 mg/L PA conditions were shortened to the 720-minute sampling, while the 500 mg/L condition showed no significant change in concentration after 240 min. These results suggest that, even in the presence of anthocyanins, ethanol seemingly influences PA adsorption kinetics; this is consistent with previous work performed in the absence of anthocyanins [7].

Lastly, under the conditions of 15%EtOH-30 °C (Figure 1e), equilibrium for the 500 mg/L PA was noted after 120 min, while the 1000 mg/L, and 1500 mg/L PA conditions continued to show significant PA loss up to the 480-minute mark. These results further suggest a synergistic influence of PA concentration, temperature, and ethanol concentration on the kinetics of PA adsorption to CWM.

### 2.2. Relative Proanthocyanidin Adsorption

**Figure 2:**
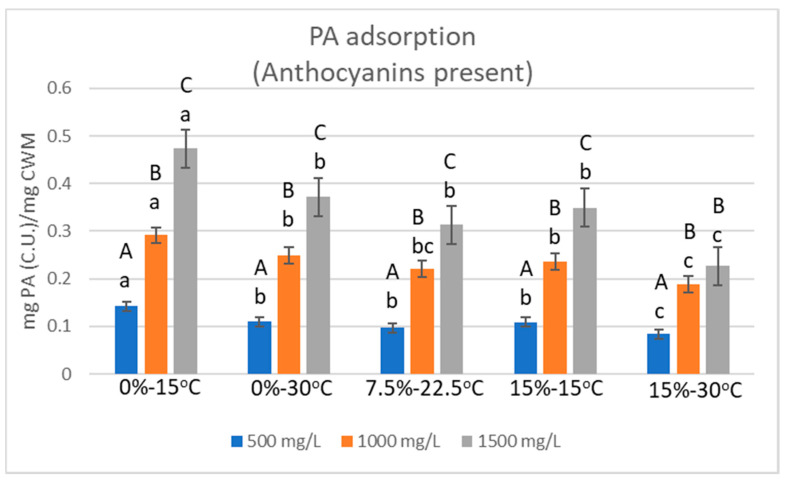
Average mass of PA adsorbed after 1440 min of contact with CWM relative to the mass of CWM (*n* = 3). Capital letters = significant difference within the same EtOH–temperature condition. Lowercase letters = significant difference between EtOH–temperature conditions of the same starting PA concentration. Significant difference defined as *p* < 0.05.

The average adsorption of PA to CWM was calculated by the difference in PA-anthocyanin solution concentrations between control and trial samples after 1440 min of exposure to CWM. Figure 2 displays the mass of PA adsorbed relative to the mass of CWM in each condition. As temperature and ethanol increased within the system, the percentage of PA adsorbed was reduced by comparison to the low-temperature, ethanol-free condition. However, the extent of adsorption for conditions containing the same starting PA concentration was not significantly different between the 0%EtOH-30 °C, 7.5%EtOH-22.5 °C, and 15%EtOH-15 °C conditions. PA adsorption in the 15%EtOH-30 °C condition was distinguished from the rest of the trials of the same starting PA concentrations, showing markedly lower adsorption per mass of adsorbent material. However, the level of PA adsorption noted in the 1000 mg/L and 1500 mg/L concentrations under this high temperature–high ethanol condition was not significantly different from one another.

For most conditions, increase in the initial concentration of PA in solution led to significant increases in the amount of PA bound to CWM; however, the percentage of available PA lost to adsorption was often quite similar within the same temperature and ethanol conditions. In the 0%EtOH-15 °C trials, the average percent decrease in PA concentration from solution was 64.3% (±4.6%), 64.7% (±3.8%), and 70.1% (±1.5%) for the 500 mg/L, 1000 mg/L, and 1500 mg/L PA conditions, respectively. Under conditions of 0%EtOH-30 °C, average decreases of 48.9% (±3.8%), 55.3% (±2.3%), and 55.1% (±1.0%) of PA in solution were noted for 500 mg/L, 1000 mg/L, and 1500 mg/L PA conditions, respectively. In the 7.5%EtOH-22.5 °C condition, PA decreased by 42.8% (±2.7%), 49.0% (±0.9%), and 46.4% (±0.3%) in solution in the 500 mg/L, 1000 mg/L, and 1500 mg/L PA conditions, respectively. Under conditions of the 15%EtOH-15 °C, PA decreased by an average of 48.3% (±5.1%), 52.4% (±1.5%), and 51.7% (±2.0%) in 500 mg/L, 1000 mg/L, and 1500 mg/L PA trials. Lastly, the 15%EtOH-30 °C condition showed that PA decreased on average by 47.3% (±1.4), 41.8% (±0.8%), and 37.3% (±1.15%) in 500 mg/L, 1000 mg/L, and 1500 mg/L PA conditions.

### 2.3. Adsorption Isotherms and Models

**Figure 3:**
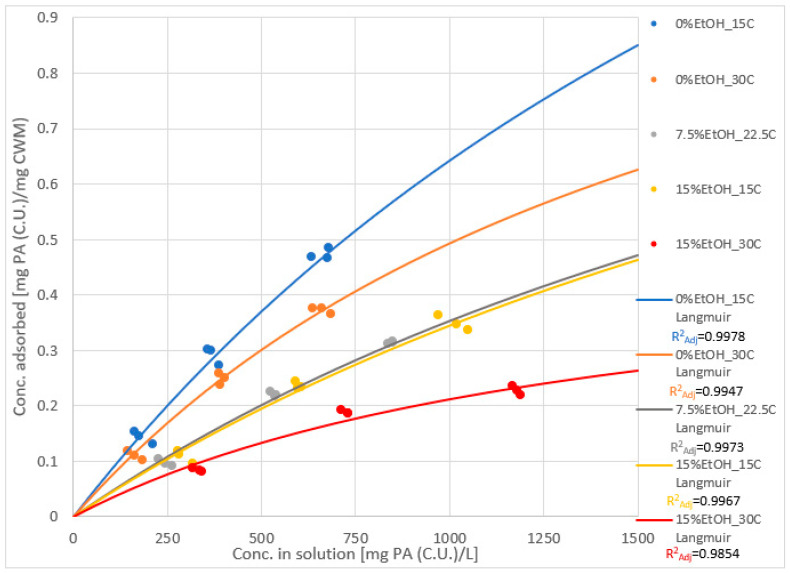
Adsorption isotherm of all experimental conditions, displayed as data points, with Langmuir models applied (*n* = 9 for each isotherm).

Figure 3 displays a PA adsorption isotherm, using the data taken from each experimental condition after 1440 min of contact with the CWM. Points on the graph represent experimental data points, whereas the solid line expresses a Langmuir model curve (Equation (2), see Materials and Methods) applied to each dataset, derived via nonlinear regression analysis. In addition, the theoretical values of the equilibrium constant (K_eq_) and the saturation point (S_CWM_) that were calculated using this technique are shown in Table 1. The visualization of the synergistic impact of temperature and ethanol on PA adsorption in the presence of anthocyanins is made even more apparent from this display.

Comparing the calculated values of K_eq_ and S_CWM_ of each condition (Table 1), a general increase in the value of the equilibrium constant and a general decrease in the saturation point constant were noted as the temperature and ethanol increased. These trends are logical considering the net extraction of PA into solution—a competing interaction to adsorption—has been shown to increase at higher temperature and in higher ethanol matrices [15,16,17]. The increasing value of K_eq_ likely reflects the reduction in the overall concentration of thermodynamically stable binding sites on the adsorbent material as temperature and ethanol increase in the system, not to be confused as an indication of increases in the concentration of PA–CWM complexes (Equation (1)).

(1) $$K_{\mathrm{eq}}=\frac{\left[{\mathrm{PA}}_{\mathrm{bound}}\right]}{\left[{\mathrm{PA}}_{\mathrm{free}}\right]\left[{\mathrm{CWM}}_{\mathrm{open}\;\mathrm{binding}\;\mathrm{site}}\right]}$$

The general decrease in S_CWM_ reflects the lower saturation point of the CWM material. This is likely the result of fewer accessible binding sites due to greater stability of PA molecules remaining in solution as opposed to forming CWM complexes at higher temperature and ethanol concentrations. This too is expected, as the comparative stability of PA-CWM complexes, compared to free PA in solution, would decrease at higher temperature and ethanol concentrations. It should be noted that the isotherm data of 7.5%EtOH-22.5 °C and 15%EtOH-15 °C were markedly similar, thus their Langmuir models are nearly identical. In general, these trends are in agreement with previous studies [7,8]. However, the values of these calculated constants are significantly different from a prior study where similar conditions were investigated but in the absence of anthocyanins [7]. This is further discussed in Section 3.3.

### 2.4. Phloroglucinolysis and GPC

Phloroglucinolysis data can be found in Table A1 in Appendix A. Comparing the trial samples (containing CWM) to the control samples (without CWM), a significant reduction in mDP and aMW was noted in all conditions. Percentage reduction in mDP and aMW ranged between 34 and 53% between trial and control samples. This result suggests a preferential adsorption of larger PA chains to the CWM. Comparing the percentage of galloylated PA in trial versus control solutions, a slight decrease was noted in most conditions. These reductions, however, were often quite small with the largest difference being approximately 8% reduction between control and trial sample in the 0%EtOH-15 °C-500 mg/L condition. The percentage of trihydroxylated B-rings (%Gallo Units) was very low for both control and trial samples, thus the data have been omitted.

**Figure 4:**
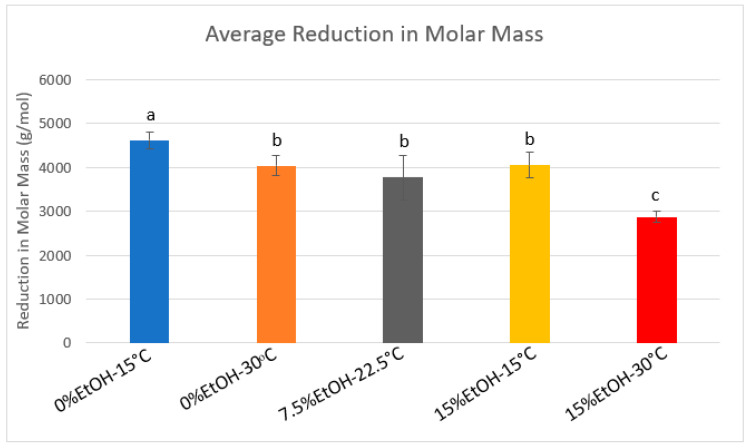
Average reduction in molar mass of PA solution due to adsorption. Values calculated from average decrease between control and trial sample molar mass (*n* = 9). Significant differences denoted by lowercase letters (*p* < 0.05).

**Figure A1:**
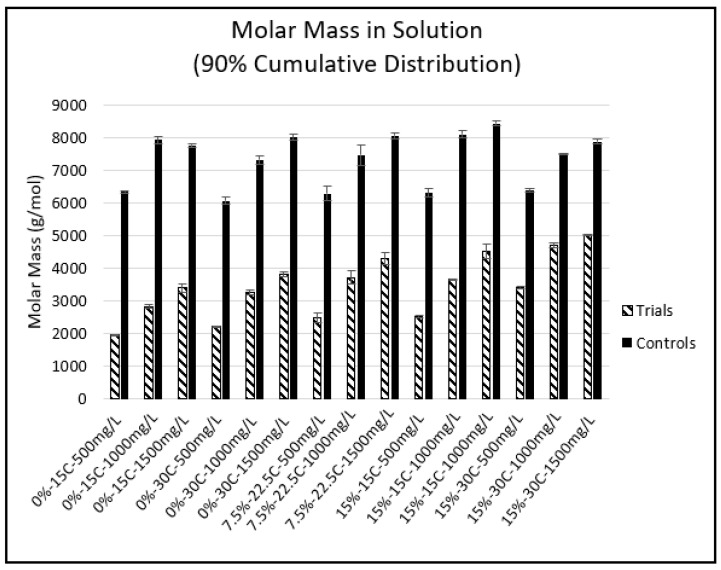
Molar mass distribution at 90% cumulative mass of control and trial samples from adsorption experiments (*n* = 3).

Gel permeation chromatography (GPC) results for each condition are shown in Figure A1 in Appendix A. In agreement with the phloroglucinolysis data, there was a noted reduction in molar mass of PA in solution between trial and control samples. Additionally, temperature and ethanol content seem to influence the sizes of PA adsorbing to the CWM. Figure 4 displays the average reduction in molar mass in the different temperature–ethanol conditions. In the absence of ethanol at low temperature, results show that the average reduction in molar mass was approximately 4622 g/mol. This equates to a nearly 63% decrease in the average PA molar mass of the original solution. In the 0%EtOH-30 °C, 7.5%EtOH-22.5 °C, and 15%EtOH-15 °C conditions, the average decreases in molar mass were 4041 g/mol (57% decrease), 3775 g/mol (52% decrease), and 4049 g/mol (53% decrease), respectively. However, these values were not significantly different from one another. Lastly, the 15%EtOH-30 °C condition showed the lowest reduction in PA molar mass in solution with an average decrease of 2873 g/mol, equating to a 40% average decrease in the control solution. These results suggest a synergistic effect of increasing temperature and ethanol impeding adsorption of larger molecular weight PA to CWM.

### 2.5. Polymeric Pigment

**Figure 5:**
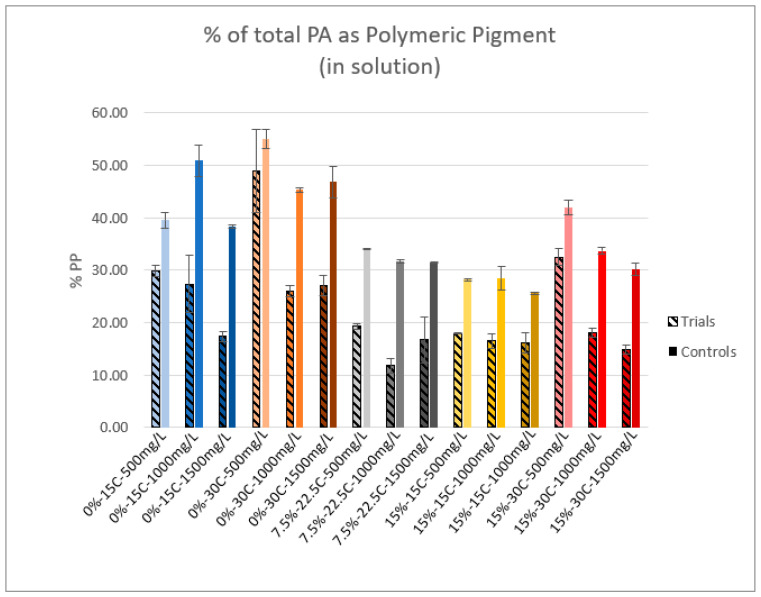
Polymeric pigment formation as a percentage of total PA in solution (*n* = 3).

Figure 5 displays polymeric pigment (PP) formation in solution as a percentage of total PA measured. In nearly all conditions, the percentage of PP in solution was reduced in the presence of CWM. Comparing the control samples of each condition, the presence of ethanol seemed to yield a lower percentage of PP formation, while increases in temperature yielded more PP formation. The 0%-30 °C-500 mg/L trial was the only one that did not show a significant difference compared to its control; however, this is likely due to its large standard deviation. A larger sample size for each condition is likely required for supporting these data and elucidating a clear trend. It should be noted that such rapid formation of polymeric pigment in the absence of acetaldehyde has not previously been reported in similar model studies. This noted phenomenon and possible explanations are further discussed in Section 3.2.

## 3. Discussion

### 3.1. Proanthocyanidin Composition

The analysis of the PA composition via phloroglucinolysis revealed a slight decrease in the percentage of galloylated PA in most conditions containing CWM. Though minor, this observation agrees with previous studies [5,18,19] and has been proposed as an artifact of the adsorption mechanism that favors PA of larger molecular weight [5]. More distinctly, the phloroglucinolysis and GPC results (Figure 4, Table A1, and Figure A1) suggest the preferential adsorption of large molecular weight PA, consistent with previous model studies containing no anthocyanins [5,7,18,20]. This is likely explained by an increased in surface area coverage of larger PA molecules to interact with more open binding sites within the CWM matrix. However, this noted preferential adsorption of large molecular weight PA in the presence of anthocyanins opposes results of a previous, 2010 study. Bindon et al. [4] noted preferential adsorption of lower molecular weight PA in the presence of pigmented PA. These conflicting results between studies may be explained by the preparation methods of the adsorbent, cell wall material. The study by Bindon et al. [4] utilized marc material from ripe, red grapes to prepare the CWM. Despite the preparatory methodology, this may have yielded a CWM with polyphenols pre-adsorbed within the matrix, influencing the mechanism of PA adsorption during adsorption experiments. This theory is supported by the fact that detectable levels of PA were shown in a later study by Bindon et al. [18] when phloroglucinolysis was performed directly on the CWM. The presence of these PA within the CWM matrix may explain the observed alteration in preferential adsorption of PA size if potential binding sites were occupied by high molecular weight PA prior to exposure of the PA solutions to the adsorbent material.

In an attempt to avoid adsorption of polyphenols to the CWM prior to mixing with the PA–anthocyanin solutions, Thompson seedless grapes were specifically chosen as a CWM source in this study due to the absence of anthocyanins and a naturally low PA content. Analysis of the CWM in this study showed no detectable levels of PA within the material (data not shown). Thus, the authors hypothesize that preferential adsorption of high molecular weight PA to grape CWM is likely to occur with or without the presence of anthocyanins.

### 3.2. Polymeric Pigment

A 2005 review by Sacchi et al. [16] reveals comparable trends between the PP analyses of this investigation and several winemaking studies. The temperature of the system was positively correlated with PP formation, while ethanol concentration exhibited a negative correlation. With regard to the comparable reduction in PP in the presence of CWM, the authors pose two potential mechanistic hypotheses. The first hypothesis is that the adsorption of free PA and anthocyanins left fewer molecules of each in the solution to react, thus the control samples formed more PP based on available reactants. The second hypothesis is that the control samples represent the estimate PP within the entire system, thus the reduction in PP in trial samples indicates preferential adsorption of PP to CWM. The latter hypothesis can potentially explain the notable increase in the amount of PA that was adsorbed compared to previous work that contained no anthocyanins [7]. So-called “Tannin-Anthocyanin” and oxidized “Anthocyanin-Tannin” polymeric pigments retain the cationic charge on the oxygen atom of the anthocyanin C-ring [21]. Potentially, ionic PP would participate in ion-dipole interactions, hydrogen bonding, Van der Waals forces, and hydrophobic interactions simultaneous. This would optimize the thermodynamic stability of the PP–CWM complex [22].

In a recent study by Teng et al. [23], large molecular weight PAs were shown to form PP more rapidly than smaller molecular weight species which seemingly supports the results of this study. However, the extent of PP formation seen in both trails and controls is incredibly rapid by comparison to works by Teng et al. and Dallas et al. [23,24]. Additionally, it has been suggested that rapid PP formation is highly dependent upon the presence of acetaldehyde [23,24,25,26], which was not present in the model wine matrix of this study. However, the concentration of PA and anthocyanins used in these adsorption experiments was at least ten times higher compared to the aforementioned previous studies. This high concentration of phenolic material, chosen based on reported concentrations in grapes, may explain the noted increase in PP after only twenty-four hours. Further study is needed to support this hypothesis.

### 3.3. Proanthocyanidin Adsorption: Presence vs. Absence of Anthocyanins

Analogous in design to the presented study, PA adsorption to grape-derived CWM was previously conducted in the absence of anthocyanins [7]. Both the study presented here and the aforementioned [7] utilized identical methods of PA isolation, PA quantification, CWM isolation, incubation temperatures, ethanol concentrations of model wine, PA concentrations, GPC methodology, and phloroglucinolysis methodology. Comparison of the results of the current study in relation to the previous work [7] yields some notable observations, emphasizing the impact of anthocyanins on PA adsorption to CWM within the model systems. The following discussion displays the respective datasets side-by-side for clear comparison.

Both studies showed similar trends to support a biphasic adsorption theory, as an initial, rapid adsorption of PA was noted followed by a slow progression to equilibrium. By contrast, the presence of anthocyanins resulted in an elongation of equilibration time for nearly all conditions containing 1000 mg/L and 1500 mg/L PA. Conditions containing 500 mg/L PA showed comparable equilibration times regardless of anthocyanin presence. However, the amount of PA material adsorbed was significantly greater in the presence of anthocyanins in the 1000 mg/L and 1500 mg/L conditions making the approximate reaction rates markedly similar between the two studies.

**Figure 6:**
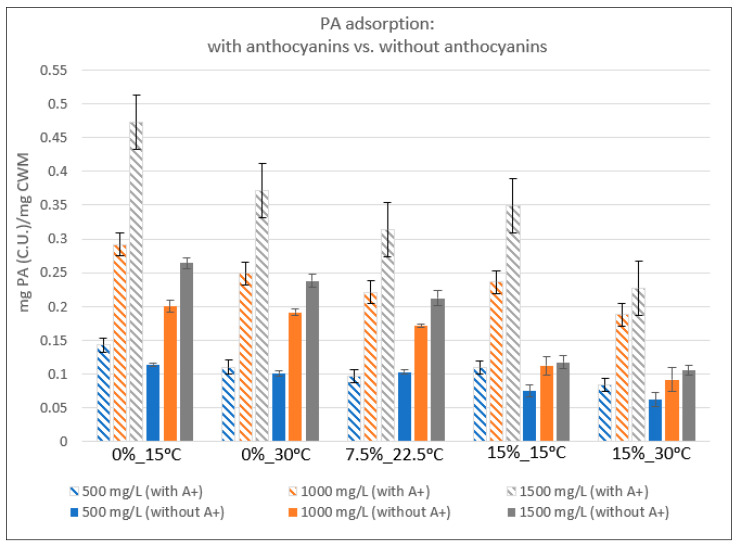
Relative PA adsorption to CWM under variable temperature and ethanol in the presence or absence of anthocyanins (*n* = 3). “A+” = anthocyanins. All “Without A+” values were derived from a previous study [7]. “A+” = anthocyanins.

**Figure 7:**
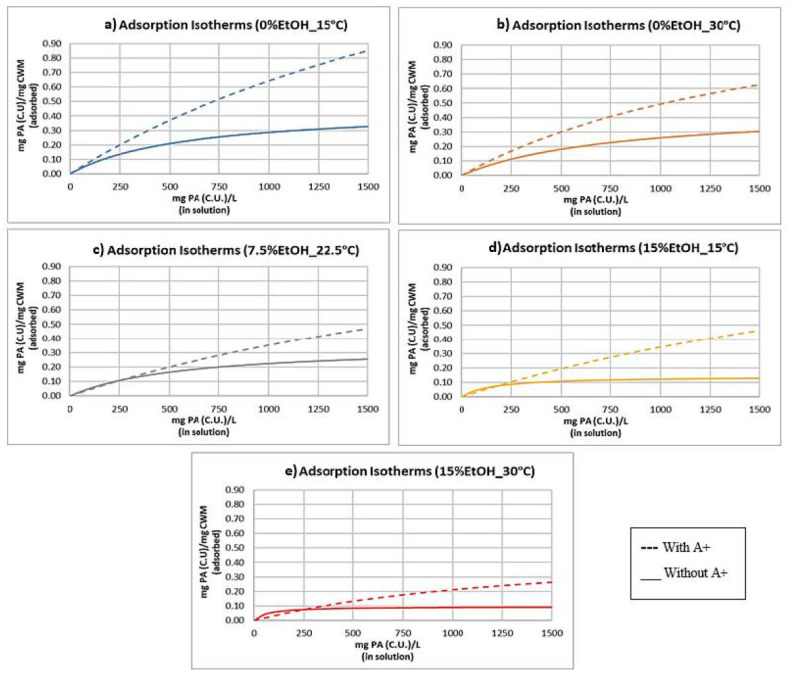
Adsorption isotherm of all conditions with Langmuir models applied (*n* = 9 for each isotherm). All “Without A+” isotherms were derived from a previous study [7]. (**a**) 0% ethanol MW, 15 °C incubation; (**b**) 0% ethanol MW, 30 °C incubation; (**c**) 7.5% ethanol MW, 22.5 °C incubation; (**d**) 15% ethanol MW, 15 °C incubation; (**e**) 15% ethanol MW, 30 °C incubation. “A+” = anthocyanins.

Figure 6 displays the quantitative results of PA adsorption to CWM measured at equilibrium, comparing the impact of having anthocyanins present in solution. The overall trends of PA adsorption related to changes in temperature, ethanol, and PA concentration are similar in most instances regardless of anthocyanin presence. The calculated values of K_eq_ were the largest under high temperature and ethanol conditions, while S_CWM_ decreased as temperature and ethanol increased within the system. However, the relative trends of the 7.5%EtOH-22.5 °C and 15%EtOH-15 °C conditions differ significantly in the comparison of this study and the previous published investigation [7]. In the absence of anthocyanins, there is a significant decrease between these two conditions with the level of PA adsorption in 15%EtOH-15 °C being more similar to that of the 15%EtOH-30 °C condition. In the presence of anthocyanins, however, the distinction between 7.5%EtOH-22.5 °C and 15%EtOH-15 °C is statistically insignificant. This supports a hypothesis for a synergistic effect of temperature, ethanol, and anthocyanin concentration on the extent of PA adsorption. This is further emphasized by a comparison of the adsorption isotherms of each study, shown in Figure 7.

In the absence of anthocyanins, there is a distinct separation in the adsorption isotherms and Langmuir models of the 7.5%EtOH-22.5 °C (Figure 7c) and 15%EtOH-15 °C (Figure 7d) conditions [7]. In the presence of anthocyanins, the data points and models of these two conditions nearly overlap (Figure 3). Additionally, the models constructed in the absence of anthocyanins display the phenomenon of adsorbent saturation (lower S_CWM_) much more readily than those constructed with anthocyanins present. Though the changes in temperature and ethanol concentration still resulted in separation between most of the Langmuir models, the adsorbent capacity value increased significantly in the presence of anthocyanins, as evident in Figure 7. This could, however, be due to an alteration in the PA adsorption mechanism. In the absence of anthocyanins, PAs clearly seem to follow Langmuir model assumptions of single-layer adsorption. Anthocyanins, however, have been shown to have some characteristics of multi-layer adsorption likely due to their ability to participate in self-association via π–π stacking [14]. It is possible that free anthocyanins and polymeric pigment promote multilayer adsorption of PA, increasing the overall adsorptive capacity of the CWM. However, an exponential increase in adsorption as PA concentration increased—a characteristic feature of BET adsorption model systems—is not noted in the tested PA concentration range. Further study is required to support such a hypothesis.

**Figure 8:**
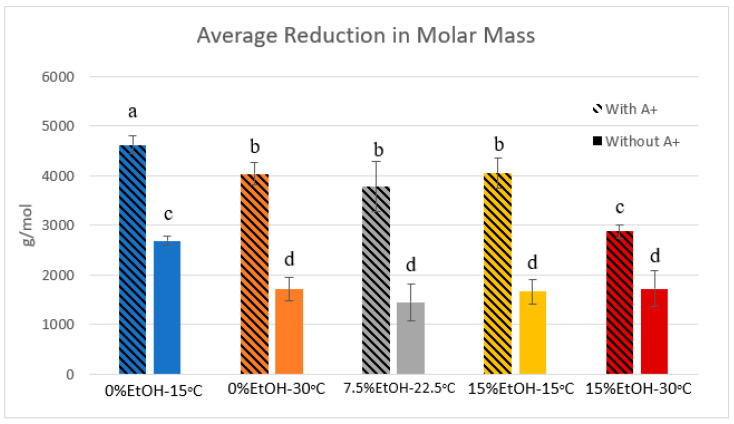
Average reduction in molar mass of PA solutions due to adsorption. Values calculated from average decrease between control sample Molar Mass and trial sample Molar Mass (*n* = 9). All “Without A+” values were derived from a previous study [7]. Significant differences denoted by lowercase letters (*p* < 0.05). “A+” = anthocyanins.

Figure 8 compares the average reduction in the molar mass of the PA solutions with and without anthocyanins present. In every tested condition, trials containing anthocyanins showed a higher reduction in molar mass compared to their respective control samples. Though increasing temperature and ethanol reduces the impact of this preferential adsorption, the 15%EtOH-30 °C condition containing anthocyanins showed approximately a 15% larger reduction in the molar mass of the PA solution compared to the same condition without anthocyanins. This ultimately suggests that the presence of anthocyanins exacerbates the preferential adsorption of large molecular weight PA. Teng et al. [23] reported that an increase in PA size is positively correlated with faster PP formation. This may potentially explain the comparative increase in both the quantitative adsorption of PA and the larger reduction in molar mass, though further study is required to support this hypothesis.

## 4. Materials and Methods

### 4.1. Model Wine Preparation

Koptec brand ethanol (95%) was purchased from Decon Laboratories, Inc. (King of Prussia, PA, USA). Deionized water was prepared in-house to a final purity of 18.2 MΩ. The model wines of varying ethanol concentrations all contained 5 g/L potassium bitartrate and were adjusted to a pH of 3.5 using hydrochloric acid.

### 4.2. Instrumentation and Software

A Genesis 10S UV-Vis Spectrophotometer (Thermo Fischer Scientific, Waltham, MA, USA) was utilized for PA quantification, and polymeric pigment analysis. An Agilent 1260 Infinity HPLC (Agilent Technologies, Santa Clara, CA, USA) equipped with a diode array detector and a 6430 triple quadrupole system was used to analyze phloroglucinolysis reaction products and analyze anthocyanin samples. An Agilent Poroshell 120 SB-C18 HPLC column (4.6 × 150 mm, 2.7 µm) was equipped for the phloroglucinolysis method. An Agilent Poroshell-120 C18 column (2.1 × 50 mm, 2.7 μm) was used for quantitation of anthocyanin samples. The same model Agilent HPLC equipped with only a diode array detector was utilized for gel permeation chromatography (GPC) analysis. An Agilent OligioPore^®^ column (7.5 × 300 mm, 6 µm) and an Agilent MesoPore^®^ column (7.5 × 300 mm, 3 µm) were connected in series for the GPC method. Instrument control of the HPLC was performed using MassHunter^®^ software. Phloroglucinolysis and anthocyanin analysis were performed using Agilent CDS ChemStation^®^, and WinGPC^®^ software (Polymer Standards Service, Philadelphia, PA, USA) was utilized for the GPC analysis.

### 4.3. Statistical Analysis

The significant difference between the datasets was determined from triplicate samples by means of one-way analysis of variance (ANOVA) followed by Tukey’s post-hoc test. A significance level of α = 0.05 was chosen for each test, thus the significant difference between datasets was defined by *p* < 0.05.

### 4.4. Isolation of Grape Skin Cell Wall Material

Skin-derived CWM was isolated from Thompson Seedless grape skins due to its lower phenolic content as well as availability year-round. CWM was prepared using a modified method from Vidal et al. [27]. Skins were manually separated from the flesh and ground under liquid nitrogen using an analytical mill (A11 Basic, IKA Works, Inc., Wilmington, NC, USA). The resulting material was extracted with 70% aqueous acetone (*v*/*v*) overnight at 4 °C to remove polyphenolics. This mixture was subsequently vacuum filtered (Whatman™ 1001-125 Grade 1 Qualitative Filter Paper, Diameter: 12.5 cm, Pore Size: 11 µm) to separate the solution from the solid material. The solids were washed with 70% aqueous acetone (*v*/*v*) until clear, followed by a deionized water wash. The solids were then extracted with 40 mM HEPES buffer (pH = 7) for one hour at room temperature in order to remove soluble polysaccharides. The mixture was again vacuum-filtered and washed with deionized water then pure acetone to remove residual HEPES buffer. The obtained solids were extracted with buffered phenol for 30 min at room temperature to remove cytoplasmic proteins. The mixture was vacuum filtered, washed with water, and subsequently washed with 80% aqueous ethanol (*v*/*v*) to remove residual phenol buffer. The resulting residue was then extracted with 50% chloroform in methanol (*v*/*v*) for 30 min at room temperature to remove lipids. The mixture was vacuum filtered again and washed with water and pure acetone to remove residual chloroform and methanol. The resulting solid material was pushed through a 500 µm mesh and finally stored under nitrogen at −20 °C until utilized.

### 4.5. Isolation of Proanthocyanidins

PAs were isolated from the skins and seeds of pre-veraison Cabernet Sauvignon grapes from Napa, CA. Skins and seeds were manually separated from the rest of the berry and separately homogenized in 70% aqueous acetone with 0.1% Trifloroacetic acid (TFA) (*v*/*v*) using a T18 digital ULTRA-TURRAX^®^ blender (IKA^®^ Works, Inc., Wilmington, NC, USA). Solutions were left to extract overnight at 4 °C. Mixtures were then centrifuged at 3220× *g* for 15 min, and the supernatant was drawn off. These solutions were concentrated under reduced pressure by rotoevaporation at 34 °C to remove acetone. The resulting aqueous solutions were then loaded onto separate low-pressure chromatography (LPC) columns of Toyopearl HW-50F (500 mL bed volume). The LPC method used for the isolation of proanthocyanidins is described by Kennedy et al. [28]. The eluates from the LPC columns containing isolated PA were concentrated under reduced pressure, lyophilized, and stored at −80 °C until utilized.

### 4.6. Isolation of Anthocyanins

**Figure 9:**
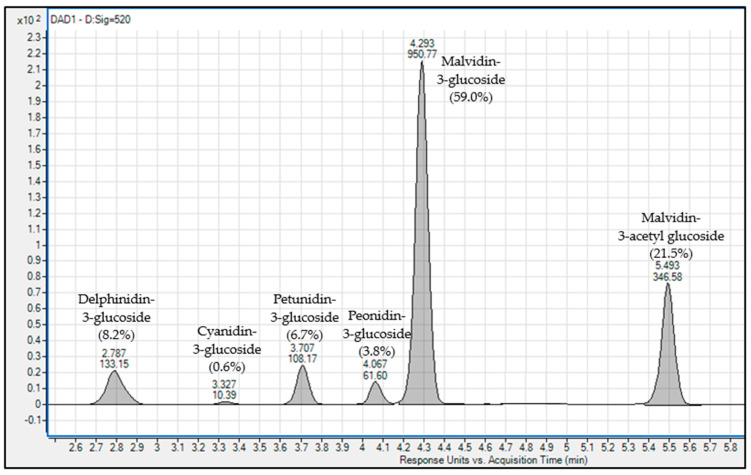
Chromatogram (520 nm) of anthocyanin in control samples. Peaks identify the six monomeric anthocyanins found in the purified anthocyanin material and their relative abundance within the mixture (quantified in “Malvidin Equivalents”).

Ripe Cabernet Sauvignon grape skins from Napa, CA were ground (T18 digital ULTRA-TURRAX^®^, IKA^®^ Works, Inc., Wilmington, NC, USA) and extracted overnight at 4 °C with 50% acidified methanol with 0.1% TFA (*v*/*v*). The methanolic extractions were then centrifuged at 3220× *g* for 15 min, and the supernatant was drawn off. These crude anthocyanin solutions were concentrated under reduced pressure by rotoevaporation at 34 °C to remove methanol. The aqueous, crude anthocyanin extracts were applied to a Toyopearl HW-50F LPC column (bed volume of 500 mL) and washed with two bed volumes of aqueous 0.1% TFA (*v*/*v*) solution in order to remove salts and oligosaccharides. Next, monomeric anthocyanins were eluted using 30% aqueous methanol with 0.1% TFA (*v*/*v*). The eluate was concentrated, and the purity was determined using a previously published RP-HPLC method [29]. The extract was concentrated under reduced pressure, lyophilized and stored at −80 °C. The individual anthocyanins of the isolated material were identified as petunidin-3-glucoside, peonidin-3-glucoside, malvidin-3-glucoside, delphinidin-3-glucoside and malvidin-3-acetylglucoside. The purity of the anthocyanin material was always over 99%. Sample chromatogram of anthocyanin isolate shown in Figure 9.

### 4.7. In Vitro Adsorption Experiments

Ten milligrams (+/− 0.5 mg) of CWM was manually weighed into 5 mL Eppendorf tubes. This mass was used in order to represent the common mass of skin CWM in a single berry [30]. Lyophilized skin and seed PA material were manually weighed and solubilized separately in model wine then mixed in a 1:1 ratio. Anthocyanin material was manually weighed, solubilized in model wine, and added to the PA mixture. Solutions contained PA concentrations of either 500 mg/L, 1000 mg/L, and 1500 mg/L as well as 500 mg/L anthocyanin material in each. PA concentrations were chosen for these experiments considering concentrations of proanthocyanidins in grapes are typically reported to be in the range of 0.5–1.5 g/L [21]. The anthocyanin concentration was chosen based on approximate concentrations commonly found in high colored red wines [31,32,33]. Experiments were conducted under varying conditions of incubation temperature and ethanol concentration (Table 2). Separately, the hydrated CWM and PA–anthocyanin solution were placed into the incubator shaker set at the experimental temperature for 1 h prior to initial contact. This allowed the CWM and solution to reach the designated temperature prior to mixing. After this initial incubation, 4.5 mL of the PA–anthocyanin solution was pipetted over the bed of CWM, and incremental sampling of the supernatant began. At t = 1, 5, 30, 60, 120, 240, 480, 720, and 1440 min, a 100 µL sample was taken from the solution in order to construct a progress curve for loss of PA from solution. These samples were stored at −80 °C until analyzed using the ferric chloride assay. In addition, a sample of the original PA–anthocyanin solution was saved to serve as a t = 0 sample. For each unique condition, adsorption experiments were performed in triplicate. Control samples containing the same PA–anthocyanin solution without CWM were also prepared in triplicate. Controls were monitored as a reference for calculating loss of PA due to reactions other than adsorption to CWM. After 1440 min, a 1.5 mL sample was taken from the supernatant of all trial and control samples and stored at −80 °C. These samples were used to analyze the qualitative impact of adsorption on PA composition in solution as well as polymeric pigment formation. All samples were analyzed within 30 days of storage to avoid degradation.

### 4.8. Quantitative Analysis of Proanthocyanidins

Quantitative analysis of the PA samples was performed using the ferric chloride assay for total iron-reactive phenolics [34]. This method was chosen as it is capable of binding all forms of PA but does not react with anthocyanins [35]. In the model systems of this study, the ferric chloride assay is likely a more appropriate quantification method than the traditionally used phloroglucinolysis or thiolysis methods, as these have shown to be unable to fractionate A-type PA as well as yield poor mass conversion in the presence of high anthocyanin content [4]. A calibration curve was constructed using (+)-catechin hydrate as a standard, thus the units of the PA samples are specified as “mg Catechin Units” (C.U.). Samples were thawed and vortexed to ensure resolubilization of any PA that may have precipitated during storage. A 75 µL aliquot of each sample was transferred to a 1 mL cuvette and mixed with 800 µL of resuspension buffer (50 g/L sodium dodecyl sulphate in 5% triethanolamine-95% deionized water, pH = 9.4). An initial reading of this mixture at 510 nm was taken to account for background absorbance. A 125 µL aliquot of ferric chloride reagent (2.7 g/L ferric chloride, 800 µL/L hydrochloric acid (37%) in deionized water) was added to the cuvette and vortexed. Absorbance of the mixture was measure at 510 nm, and the concentration of the PA solution at each timepoint was determined against the (+)-catechin calibration curve. The amount of PA adsorbed to the CWM was determined by difference between the concentration of the trial samples from the concentration of the control samples.

### 4.9. Adsorption Isotherms

For each trial sample, the concentration of PA in solution and the concentration of PA adsorbed to the CWM at equilibrium was determined via the ferric chloride assay. These data were plotted in an adsorption isotherm for comparison. Previous studies of PA in model wine systems absent of anthocyanins suggested that PA–CWM interactions follow Langmuir model trends [7,8]. Thus, a Langmuir adsorption model curve was applied to each condition via regression analysis of the data to the Langmuir Equation (Equation (2)). Calculated constants were compared to previous results.

(2) $$q_{\mathrm{PA}}=\frac{C_{\mathrm{PA}}\times K_{\mathrm{eq}}\times S_{\mathrm{CWM}}}{1+C_{\mathrm{PA}}\times K_{\mathrm{eq}}}$$

q_PA_ = mass of proanthocyanidin adsorbed per mass of CWM at equilibrium (mg PA (C.U.)/mg CWM);
C_PA_ = the concentration of proanthocyanidins in solution at equilibrium (mg PA (C.U.)/L);
K_eq_ = the equilibrium constant of the system (L/mg PA (C.U.));
S_CWM_ = the saturation point of the CWM (mg PA (C.U.)/mg CWM).

### 4.10. Qualitative Analysis of Proanthocyanidin–Anthocyanin Solutions

Supernatant samples taken at the end of the adsorption experiments were separated into three different fractions. The first of these fractions—to be used for phloroglucinolysis—was lyophilized, and the resulting material was manually weighed and solubilized in 125 µL of acidified methanol (0.1 N HCl). Phloroglucinolysis was performed by reacting 50 µL of each solution with 50 µL of phloroglucinol reagent at 50 °C for 20 min. The reaction was quenched with 0.5 mL of sodium acetate (40 mM). Samples were centrifuged, transferred to amber glass vials, and analyzed via HPLC-DAD-MS/MS using a previously defined method [28]. Phloroglucinolysis was performed in duplicate for each sample. A calibration curve using (+)-catechin as a standard was constructed. Molar absorptivities of epicatechin, epicatechin gallate, epigallocatechin, and their phloroglucinol adducts were applied to the catechin calibration curve to construct individual calibrations for each species [28]. Mean degree of polymerization (mDP), average molecular weight (aMW), %galloylation, and %gallo units of each sample were calculated from this method.

The second fraction of each sample was likewise lyophilized and solubilized in 0.5 mL of GPC mobile phase (0.15 M lithium chloride, 5% deionized water, 1% glacial acetic acid, 94% *N*,*N*-Dimethylformamide). The Agilent OligioPore^®^ and MesoPore^®^ columns were connected in series to the HPLC and equilibrated in the GPC mobile phase for 1 h at a flow rate of 0.65 mL/min prior to the first injection. Then, 20 µL of each sample was injected, and the isocratic flow of the mobile phase was maintained at a rate of 0.65 mL/min for 40 min. WinGPC^®^ software was used to analyze the cumulative molar mass distribution of each sample. Standards for constructing a calibration curve were isolated from pre-veraison Cabernet Sauvignon skin and seed material using a previous developed method [36]. Molar mass at 90% cumulative distribution was derived for each sample using this method.

The third fraction taken from each sample was utilized for polymeric pigment (PP) analysis, using a previously defined method [37]. Frozen samples were thawed and vortexed to ensure resolubilization of any precipitated material. In a 2 mL cuvette, 500 µL of the PA-anthocyanin sample was combined with 1 mL of washing buffer (0.17 M sodium chloride, 1.2% glacial acetic acid in deionized water, pH = 9.4) and 120 µL of bleaching reagent (0.36 M potassium metabisulfite in deionized water). Absorbance at 520 nm was recorded using a UV–Vis spectrophotometer. Then, another 500 µL sample of each sample was transferred to a 1.5 mL Eppendorf tube and combined with 1 mL of Bovine Serum Albumin (BSA) solution (1 mg/mL BSA in washing buffer). After incubation at room temperature for 15 min, the sample was centrifuged at 25,200× *g* for 5 min, and 1 mL of the resulting supernatant was transferred to a 2 mL cuvette. Eighty microliters of bleaching reagent was added to this cuvette, and absorbance at 520 nm was again recorded by the spectrophotometer. These two absorbance values, respectively, correspond to bleachable small polymeric pigment (SPP) and unbleachable large polymeric pigment (LPP) [37]. Due to the potential diversity of chemical structures, this method is limited to describing polymeric pigments as relative abundance by comparison of the absorbance units (AU) of the respective samples. Thus, the average percent of polymeric pigment formation in solution was calculated for each sample from the ratio of polymeric pigment AU to total iron reactive phenolic AU. It should be noted that these calculated values likely contain some inherent error due to the comparison of AU values of polymeric pigment being measured at 520 nm, while iron reactive phenolics were measure at 510 nm. This error is assumed to be minimal.

## 5. Conclusions

In all conditions, PA adsorption to CWM decreased as temperature and ethanol concentration increased within the system. Preferential adsorption of large molecular weight species was noted in all conditions, but the reduction in molar mass of the solution was significantly less at high temperature and high ethanol conditions. The presence of anthocyanins seemingly increased the extent of PA adsorption in all conditions compared to similar adsorption experiments in the absence of anthocyanins. Larger reductions in molar mass of the PA solution were also noted. This increase in PA adsorption is potentially explained by the rapid formation of PP noted in the model solution. As discussed, PP would likely yield more stable complexes with the CWM or possibly multi-layer adsorption through π–π stacking of anthocyanins. The higher than expected rate at which PP formed in each system is potentially due to the use of high concentrations of both PA and anthocyanins in the model solutions of this study, though further study is needed to confirm this hypothesis.

In the context of red wine production, the results of this study suggest a preferential loss of PP to cap CWM compared to non-pigmented PA and free anthocyanins. Considering that PP helps to stabilize the color during bottle aging and is believed to impart a beneficial mouthfeel characteristic to red wines, retention of these compounds in solution is desirable. Thus, understanding and predicting their fate are highly applicable. Additionally, the results of this work suggest a significant quantitative and qualitative impact of anthocyanins on PA adsorption. This, in turn, reveals that anthocyanin concentration should be considered when attempting to model phenolic extraction of red wine fermentations.

Future investigations will seek to describe the quantitative and qualitative change in anthocyanin content analyzed during this study. This may yield further mechanistic understandings of polyphenol adsorption to CWM or PP formation and help improve the accuracy of computation phenolic extraction models. In addition, future studies will seek to specifically describe the effects of PA and anthocyanins concentration, PA size, temperature, and ethanol concentration on the kinetics of PP formation in the absence of acetaldehyde. In conjunction with previous works, this will yield a fuller understanding of phenolic extraction during the winemaking process.

## Figures and Tables

**Table 1:** Langmuir equation constants for each experimental condition. Values derived via regression analysis of experimental data fit to the Langmuir equation.

Experimental Condition	K_eq_	S_CWM_
0%EtOH-15 °C	3.63 × 10^−4^	2.41
0%EtOH-30 °C	5.70 × 10^−4^	1.36
7.5%EtOH-22.5 °C	3.28 × 10^−4^	1.43
15%EtOH-15 °C	3.02 × 10^−4^	1.48
15%EtOH-30 °C	6.98 × 10^−4^	5.14 × 10^−1^

**Table 2:** Description of adsorption experiment parameters. All experiments contained 500 mg/L anthocyanin material.

Starting Concentration of PA Solution (mg/L)	Incubation Temperature (°C)	Ethanol Percentage of Model Wine (*v*/*v*)
500	15	0%
1000	15	0%
1500	15	0%
500	15	15%
1000	15	15%
1500	15	15%
500	22.5	7.5%
1000	22.5	7.5%
1500	22.5	7.5%
500	30	0%
1000	30	0%
1500	30	0%
500	30	15%
1000	30	15%
1500	30	15%

## Abbreviations

PA	proanthocyanidin(s)
CWM	cell wall material
EtOH	ethanol
C.U.	catechin units
mDP	mean degree of polymerization
aMW	average molecular weight
PP	polymeric pigment

## Appendix A

**Table A1:** Phloroglucinolysis results from PA–anthocyanin solutions after 1440 min of incubation (*n* = 6 for each sample). * = significant difference in average measurements between control and trial samples of the same EtOH–temperature conditions (*p* < 0.05). mDP = mean degree of polymerization, aMW = average molecular weight.

Experimental Condition	Starting PA Concentration (mg/L)	mDP	mDP StDev	%Galloylation	%Galloylation StDev	aMW	aMW StDev
0%-15 °C Control	500	7.97	0.25	65.32	1.57	3084.35	82.04
0%-15 °C Trial	500	3.48 *	0.20	57.17 *	0.93	1305.49 *	77.72
0%-15 °C Control	1000	11.82	0.85	61.35	1.84	4505.64	340.39
0%-15 °C Trial	1000	5.21 *	0.40	58.44	4.9	1961.52 *	121.34
0%-15 °C Control	1500	13.99	1.37	62.92	0.85	5365.04	537.82
0%-15 °C Trial	1500	7.74 *	0.28	55.04 *	0.45	2874.32 *	107.74
0-30 °C Control	500	11.46	0.16	62.28	0.61	4383.77	66.06
0-30 °C Trial	500	4.76 *	0.14	56.16 *	0.69	1776.63 *	51.84
0%-30 °C Control	1000	14.91	0.20	60.90	0.44	5670.07	84.65
0%-30 °C Trial	1000	7.10 *	0.20	56.07 *	0.55	2646.63 *	76.83
0%-30 °C Control	1500	18.67	0.80	61.22	0.37	7109.64	313.8
0%-30 °C Trial	1500	9.39 *	0.30	56.15 *	0.66	3503.16 *	120.11
7.5%-22.5 °C Control	500	9.83	0.46	63.78	1.95	3782.41	161.68
7.5%-22.5 °C Trial	500	4.93 *	0.10	59.81 *	0.85	1869.43 *	41.9
7.5%-22.5 °C Control	1000	14.76	0.47	61.12	0.79	5617.58	195.18
7.5%-22.5 °C Trial	1000	8.26 *	0.32	59.18	0.57	3119.47 *	128.02
7.5%-22.5 °C Control	1500	17.06	0.61	61.76	0.39	6511.12	239.88
7.5%-22.5 °C Trial	1500	9.88 *	0.30	59.56	0.43	3736.88 *	118.75
15%-15 °C Control	500	10.68	0.18	61.97	0.19	4081.13	70.95
15%-15 °C Trial	500	5.35 *	0.09	60.12 *	0.43	2030.54 *	38.17
15%-15 °C Control	1000	14.72	0.54	61.74	0.53	5616.28	217.58
15%-15 °C Trial	1000	8.01 *	0.14	59.89*	0.30	3032.60 *	54.27
15%-15 °C Control	1500	18.05	0.83	62.22	0.34	6900.68	325.69
15%-15 °C Trial	1500	10.70 *	0.38	60.64 *	0.42	4065.38 *	151.15
15%-30 °C Control	500	11.24	0.26	63.70	0.77	4324.30	101.11
15%-30 °C Trial	500	6.75 *	0.17	63.88	0.35	2599.33 *	69.52
15%-30 °C Control	1000	16.90	0.39	62.04	0.20	6456.06	150.41
15%-30 °C Trial	1000	11.34 *	0.83	63.68	1.77	4360.63 *	305.77
15%-30 °C Control	1500	18.56	0.57	62.80	0.21	7111.70	220.79
15%-30 °C Trial	1500	13.07 *	0.29	62.90	0.46	5008.61 *	119.10

**Table A2:** Average mass PA adsorbed to cell wall material after system reached equilibrium in the presence of anthocyanins. Values are presented in mg PA (C.U.) per mg CWM (*n* = 3). Respective standard deviations are displayed to the right of each data point. Values are represented in Figure 2.

Condition	500 mg/L	StDev	1000 mg/L	StDev	1500 mg/L	StDev
0%EtOH-15 °C	0.1424	0.0107	0.2913	0.0169	0.4733	0.0103
0%EtOH-30 °C	0.1101	0.0085	0.2489	0.0102	0.3719	0.0067
7.5%EtOH-22.5°C	0.0964	0.0061	0.2206	0.0042	0.3135	0.0021
15%EtOH-15 °C	0.1086	0.0115	0.2359	0.0069	0.3492	0.0135
15%EtOH-30 °C	0.0840	0.0032	0.1879	0.0037	0.2272	0.0078

**Table A3:** Average mass PA adsorbed to cell wall material after system reached equilibrium in the absence of anthocyanins. Values derived from previous study [7] and represented in Figure 6. Values are presented in mg PA (C.U.) per mg CWM (*n* = 3). Respective standard deviations are displayed to the right of each data point.

Condition	500 mg/L	StDev	1000 mg/L	StDev	1500 mg/L	StDev
0%EtOH-15 °C	0.1136	0.0024	0.2000	0.0084	0.2641	0.0081
0%EtOH-30 °C	0.1007	0.0036	0.1910	0.0046	0.2381	0.0093
7.5%EtOH-22.5 °C	0.1027	0.0027	0.1707	0.0026	0.2123	0.0114
15%EtOH-15 °C	0.0748	0.0085	0.1119	0.0132	0.1174	0.0091
15%EtOH-30 °C	0.0616	0.0103	0.0910	0.0175	0.1051	0.0075

**Table A4:** Average reduction in molar mass of PA solutions due to adsorption. Values represented in Figure 8 (*n* = 9). All “Without A+” values were derived from a previous study [7]. “A+” = anthocyanins, “MM” = Molar Mass.

Condition	Average MM Reduction (With A+)	StDev	Average MM Reduction (Without A+)	StDev
0%-15 °C	4622.81	186.01	2688.07	90.33
0%-30 °C	4040.90	224.11	1713.57	231.24
7.5%-22.5 °C	3775.43	508.73	1444.71	362.84
15%-15 °C	4049.18	296.20	1659.02	247.51
15%-30 °C	2872.66	133.12	1718.05	357.99
